# The complete mitochondrial genome of the *Sitta villosa* (Passeriformes: Sittidae) from China

**DOI:** 10.1080/23802359.2020.1773341

**Published:** 2020-06-05

**Authors:** Zhirong Zhang, Shuhui Mi, Qiuxia Guo, Zhuo Zhang, Pengfei Yan, Zhensheng Liu, Liwei Teng

**Affiliations:** a College of Wildlife and Protected Area, Northeast Forestry University, Harbin, China; b State-owned Forest in Taiyuan, Taiyuan, China; c Taiyuan Zoo, Taiyuan, China; d Key Laboratory of Conservation Biology, National Forestry and Grassland Administration, Harbin, China

**Keywords:** Mitochondrial genome, *Sitta villosa*, phylogenetic analysis

## Abstract

We describe the whole mtDNA genome of the Chinese nuthatch *Sitta villosa* in Tianlong Mountain, Shanxi, China. It is actually a circular molecular of 16,816 bp in length and consists 13 protein-coding genes, 22 transfer-RNA genes, 2 ribosomal-RNA genes, and 1 control region (D-loop, 1,243 bp in length). The nucleotide composition is 30.3% A, 30.1% C, 14.5% G, 25.1% T. The phylogenetic analysis based on the maximum likelihood method revealed the relationship of *S. villosa* was close to the three reported species within the same genus *Sitta*, which are *S. nagaensis*, *S. himalayensis* and *S. carolinensis*.

The Chinese nuthatch (*Sitta villosa*) is a small bird (approximately 11 cm in length) in the family of Sittidae, distributed in South Korea, North and North-east China (Mackinnon et al. 2000; Zheng 2017). This species dwells most extensively in the low mountain areas to subalpinal forest within coniferous forests and mixed forests (Liu and Deng 2015). Only two subspecies, both *S. villosa bangsi* (endemic to China) and *S. villosa villosa,* belong to the Chinese nuthatch. It is a beneficial bird that plays an important ecological role in forest conservation.

We sequenced the complete mitochondrial genome of the Chinese nuthatch with a fresh muscle sample collected from a natural death individual in Tianlong Mountain, Shanxi, China and stored in the College of Wildlife and Protected Area, Northeast Forestry University (No. HTS201912).

The complete mitochondrial genome of the Chinese nuthatch (GenBank accession number: MT444149) is actually a circular molecular of 16,816 bp in length, contains 1 control region (D-loop, 1,243 bp in length) and a conserved set of 37 genes including 2 ribosomal-RNA genes (12S rRNA and 16S rRNA), 22 transfer-RNA genes, and 13 protein-coding genes. The nucleotide composition is 30.3% A, 30.1% C, 14.5% G, 25.1% T. In addition to ND6 and 8 transfer-RNA genes, all other mitochondrial genes are encoded on the heavy strand (H strand). Except for ND1 and COX1 initiate with ATC, the remaining 11 protein-coding genes use ATG as the start codon. The sequence length of 22 transfer-RNA genes range from 66 bp (tRNA-Ser (gcu)) to 75 bp (tRNA-Ser (uga) and tRNA-Leu (uaa)), the 12S rRNA and 16S rRNA is 978 bp and 1597 bp in length, respectively.

The evolutionary analyses were conducted in MEGA X using the maximum likelihood method based on complete mitochondrial genomes of 21 species in Passeriformes (Figure 1) (Felsenstein 1985; Tamura and Nei 1993; Kumar et al. 2018). It revealed that *S. villosa* was close to the three reported species within the same genus *Sitta*, including *S. nagaensis*, *S. himalayensis* and *S. carolinensis*.

**Figure 1.**
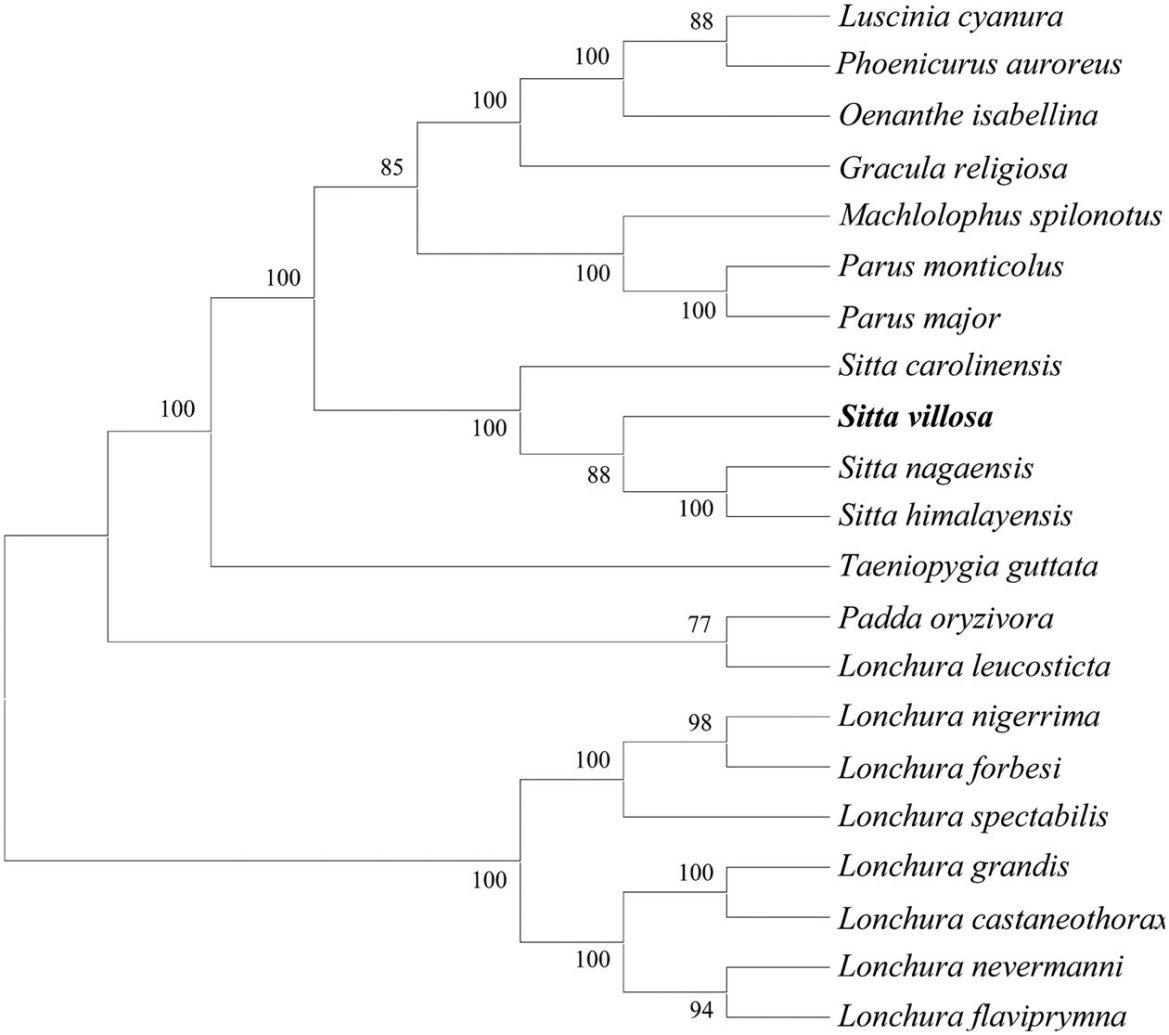
Evolutionary analysis by the maximum likelihood method based on complete mitochondrial genomes of 21 species in Passeriformes. GenBank accession numbers as follows: *Luscinia cyanura* (KF997864.1), *Phoenicurus auroreus* (KF997863.1), *Oenathe isabellina* (NC_040290.1, *Gracula religiosa* (JF937590.1), *Machlolophus spilonotus* (KX388476.1), *Parus monticolus* (KX388481.1), *Parus major* (NC_040875.1), *Sitta carolinensis* (KJ909195.1), *Sitta villosa* (this study), *Sitta nagaensis* (NC_042731.1), *Sitta himalayensis* (NC_042730.1), *Taeniopygia guttata* (DQ453515.1), *Padda oryzivora* (KT633398.1), *Lonchura leucosticta* (MF770331.1), *Lonchura nigerrima* (MF770449.1), *Lonchura forbesi* (MF770385.1), *Lonchura spectabilis* (MF770460.1), *Lonchura grandis* (MF770405.1), *Lonchura castaneothorax* (MF770362.1), *Lonchura nevermanni* (MF770441.1), and *Lonchura flaviprymna* (MF770374.1).

## Data Availability

The data that support the findings of this study are openly available in GenBank of NCBI at https://www.ncbi.nlm.nih.gov/, reference number MT444149.
